# Comparison of Landing Biomechanics in Male Amateur Basketball Players with and without Patellar Tendinopathy during Simulated Games

**DOI:** 10.5114/jhk/201318

**Published:** 2025-04-30

**Authors:** Fengping Li, Dong Sun, Yang Song, Yufei Fang, Xuanzhen Cen, Qiaolin Zhang, Yaodong Gu

**Affiliations:** 1 Faculty of Sports Science, Ningbo University, Ningbo, China.; 2 Department of Biomedical Engineering, Faculty of Engineering, The Hong Kong Polytechnic University, Hong Kong, China.; 3 Ningbo No. 2 Hospital, Ningbo, China.; 4 Doctoral School on Safety and Security Sciences, Óbuda University, Budapest, Hungary.; 5 Faculty of Engineering, University of Szeged, Szeged, Hungary.

**Keywords:** injury prevention, patellar tendon force, simulated basketball load, orthogonal partial least squares-discriminant analysis, guards

## Abstract

This study compared male amateur basketball players with asymptomatic patellar tendon tendinopathy (ASYM) to healthy controls (CON) during simulated games to explore the differences in patellar tendon force (PTF) and related metrics. Data on kinematics, kinetics, and electromyography were collected from 24 participants, comprising 12 in the ASYM group and 12 in the CON group, performing a stop-jump task in four stages (1^st^, 2^nd^, 3^rd^, 4^th^). A musculoskeletal model was used to calculate PTF, and Orthogonal Partial Least Squares Discriminant Analysis (OPLS-DA) identified significant variables. In the first three stages, the ASYM group showed significantly greater PTF and the ankle discrete relative phase (ADRP) than the CON group, with differences of 0.98, 0.79, 0.81kg•BW^−1^ (p < 0.001) and 7.34°, 11.24°, and 2.49° (p < 0.05), respectively. In the last three stages, the ASYM group had a higher knee co-activation index (KCAI) than the CON group, with differences of 0.33, 0.28, and 0.25 (p < 0.05). Correlations between PTF and the ADRP and between PTF and the KCAI were the highest, at 0.58 and 0.61, respectively. The OPLS-DA model effectively distinguished between the groups, suggesting potential applications in tendon health monitoring. The findings suggest that elevated PTF may be linked to tendinopathy in male amateur basketball players, highlighting the importance of comprehensive strategies, such as improving ankle symmetry and optimizing muscle coordination to mitigate tendon load and injury risk

## Introduction

Patellar tendinopathy is common in sports involving repetitive jumping, especially in young male athletes (15–30 yrs) (Malliaras et al., 2015). In previous research, patellar tendinopathy prevalence among elite athletes reached approximately 14%, with volleyball and basketball showing rates of 45% and 32%, respectively. Non-elite athletes showed a prevalence range from 2.5% to 14.4% (Tayfur et al., 2022). Over an eight-season period, a study conducted in a multidisciplinary sports club reported patellar tendinopathy incidence rates of 22.7% (95% Confidence Interval [CI]: 16.6–30.2) among professional basketball players and 11.4% (95% CI: 8.4–15.1) among youth players (Florit et al., 2019). In basketball, guards exhibit the highest injury rates. Given the diverse roles and positional demands in basketball, it is crucial to investigate patellar tendon (PT) injuries with a focus on specific player positions. Guards, who engage in high-intensity actions such as direction changes and jumps, are particularly susceptible to tendon, ligament, and muscle injuries (Petway et al., 2020; Torres-Ronda et al., 2022; Hidayat et al., 2025). However, in the field of basketball injury biomechanics, studies that specifically differentiate between positional roles are scarce, and targeted research on guards is especially limited.

Patellar tendinopathy significantly impacts athletes, with over one-third unable to return to sport within 6 months, and 53% being forced to retire (Cook et al., 1997). Recovery rates are unsatisfactory, with recurrence rates over 25% (Hägglund et al., 2011), and up to 50% of affected athletes ending their careers (Cook and Purdam, 2009). Despite its prevalence, the biomechanical factors contributing to patellar tendinopathy are not well understood. Altered landing kinematics have been linked to patellar tendinopathy onset (Van der Worp et al., 2014), suggesting that landing biomechanics, including kinematics and kinetics, may influence or be influenced by patellar tendinopathy. Therefore, understanding the relationship between patellar tendon force (PTF) and patellar tendinopathy could aid in identifying contributing factors and thus developing prevention strategies (Pietrosimone et al., 2020, 2021). However, since PTF is relatively difficult to measure, we have selected several more readily measurable and sport-specific indicators based on previous related studies. This will be beneficial for establishing classification and prediction models to better monitor the health status of athletes in the future (Dorris et al., 2024). Therefore, this study focuses on several key metrics such as PTF, knee joint angular velocity (KAV), knee joint stiffness (KS), hip-knee-ankle joint symmetry, and lower limb muscle activation.

Given the importance of understanding these biomechanical factors, it is crucial to investigate specific metrics that could provide deeper insights into patellar tendinopathy development and prevention. Previous studies have frequently used the drop-jump task for data collection, which differs from actual landing patterns. Hence, we have chosen stop-jumps as our testing action. Moreover, there is currently limited research focusing on the cumulative impact of game-specific loads' effects on the PT, particularly in basketball, where games consist of distinct phases (Scanlan et al., 2018). Fatigue has been demonstrated to significantly alter jump-landing biomechanics, particularly in the trunk and pelvic regions, which consequently affects lower extremity loading (Vermeulen et al., 2023). In addition, the integration of machine learning in analyzing the relationship between training load and injury risk is a new but fast-growing research area. However, there is no clear consensus on the most relevant variables for analysis (Cui et al., 2023; Majumdar et al., 2022). Orthogonal Partial Least Squares-Discriminant Analysis (OPLS-DA) is a method designed to extract information from predictor variables (X) to predict or classify samples into predefined classes or groups represented by response variables (Y). However, unlike traditional ways, OPLS-DA decomposes the variation in the data into predictive and orthogonal components. This orthogonalization step separates the variation correlated with class discrimination (predictive variation) from unrelated systematic variation (orthogonal variation), thereby enhancing the interpretability of the model and improving its predictive performance. For relevant indicators, we used machine learning methods to establish classification models, and by leveraging this statistical technique, this study aimed to gain deeper insights into the factors associated with patellar tendinopathy and develop more accurate predictive models for risk assessment and diagnosis. Thus, it would be possible to supplement cumbersome screening with more readily available indicators and to conduct timely and tracking detection in daily training and competitions.

The investigation of these variables aimed to analyze the biomechanical variances observed in landing mechanics, which might be associated with asymptomatic abnormalities in the PT structure during jump landings, and how these factors varied under accumulated game-specific simulated basketball loads. This exploration would aid in uncovering factors associated with patellar tendinopathy (Tayfur et al., 2022). Upon identifying these patellar tendinopathy-related factors, we could further utilize machine learning techniques to establish classification models for these relevant indicators. This would enable ongoing monitoring of PT health during daily training and competitions, thereby providing a more scientific basis for injury prevention and rehabilitation program development. We hypothesized that abnormalities in the PT structure would be partially associated with the proposed indicators. Furthermore, among amateur basketball players, the differences in these related indicators would vary under different accumulated loads.

## Methods

### Participants

An a priori power analysis was conducted using G*Power version 3.1.9.7 (Faul et al., 2007) to estimate the required sample size. The analysis was based on data from Pietrosimone et al. (2022) (N = 30), which compared asymptomatic and control groups on the knee-flexion angle during the stance phase of landing. The reported effect size ranged from Cohen's *d* = 0.96 to 1.01 across the early (8%–13%, Cohen's *d* = 0.99) and late (74%–94%, Cohen's *d* = 0.96) stance phases, considered to be large using Cohen's (1988) criteria. For ANOVA calculations, the effect size was converted to f = 0.505. Using a significance level of α = 0.05 and statistical power of 0.80, a total of 24 participants (12 per group) were deemed sufficient. Participants were recruited based on the following eligibility criteria: (1) being a starting basketball player without any professional training experience, and (2) competing in school- or university-level games 1–2 times per week with additional self-training sessions 1–2 times per week (Li et al., 2023). A total of 24 right-dominant male amateur basketball players were included. Dominance was determined based on the preferred leg for kicking a ball, with the non-dominant leg more frequently used for jumping and landing during layups in basketball (Benítez-Martínez et al., 2019). All participants underwent a single-legged decline squat test to evaluate PT functionality (Herrington, 2014; Levinger et al., 2006). Subsequently, a professional musculoskeletal ultrasound physician conducted bilateral knee ultrasound imaging and diagnosis for all participants. Those exhibiting PT ultrasound abnormalities (hypoechoic region ≥ 2 mm) were assigned to the asymptomatic with PT abnormality (ASYM) group, while the remaining participants were assigned to the health control (CON) group (Benítez-Martínez et al., 2019; Cushman et al., 2021). Both groups were composed of an equal number of participants, with 12 in each group. Informed consent was obtained, and the protocol was approved by the Ethics Committee of the Faculty of Sports Science, Ningbo University, Ningbo, China (protocol code: RAGH20231105; approval date: 05 November 2023).

### Experimental Protocol

Participants began with a 5-min standard dynamic warm-up, which included low-intensity jogging, whole-body dynamic and static stretches, and brief bouts of high-intensity running (Scanlan et al., 2012). They then performed two maximal-effort stop-jumps, touching a height marker. The average height determined the target for the actual stop-jump test, set at 85% of this height. After setting the target height, the Basketball Exercise Simulation Test (BEST) was used to simulate basketball-specific activity and impose game-specific simulated basketball load on the participants. High-intensity, intermittent exercise protocols, such as those employed in this study, have been shown to induce acute and prolonged neuromuscular fatigue, making them valuable tools for biomechanical screenings under fatigued conditions (Vermeulen et al., 2024).

**Figure 1:**
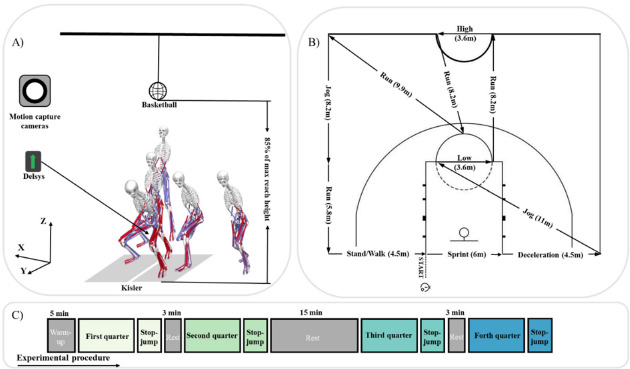
Diagram of data acquisition. (A) Diagram of the stop-jump test. (B) The schematic depiction of the Basketball Exercise Simulation Test. High indicates the high-intensity shuffling; Low indicates the low-intensity shuffling. (C) Experimental procedure.

Briefly, the BEST consisted of 4 × 10-min quarters with 3-min rest intervals between quarters, except for a 15-min rest interval at halftime to simulate a basketball game. Each circuit was time-bound (30 s) and performed continuously across each simulated quarter, with a maximum of 20 circuits per 10-min quarter. Participants typically completed each circuit within 25 s, allowing at least 5 s of rest before starting the next circuit. If participants failed to complete a circuit within the allotted time, they were required to come to a complete stop and begin the next circuit immediately (Scanlan et al., 2018). At the end of each quarter, participants underwent the stop-jump vertical reach test as shown in Figure 1A. The overall experimental procedure is illustrated in Figure 1C. Participants performed a one-step stop-jump to touch a marker on a height measurement device. Five jump-landing trials were conducted, and three successful trials were averaged for further data analysis. A successful trial required participants to touch the marker with their right hand and land with both feet on the force plate. The rest intervals were spent in a seated position. The different activity types and distances performed during each BEST circuit are shown in Figure 1B.

### Data Acquisition

Thirty-eight retroreflective markers were affixed following a previously established protocol (Song et al., 2023). Motion capture was conducted using a 10-camera Vicon system at 200 Hz (Vicon Vero, Oxford Metrics Ltd., Oxford, UK) and ground reaction forces (GRF) were simultaneously measured at 1000 Hz via two large stationary Kistler 3D force plates (model 9287C, dimensions 900 × 600 × 100 mm, Kisler Instrumente AG, Winterthur, Switzerland). Electromyography (EMG) signals from lower limb muscles including rectus femoris (RF), vastus lateralis (VL), vastus medialis (VM), biceps femoris (BF), semitendinosus (ST), medial gastrocnemius (MG), peroneus longus (PL), and tibialis anterior (TA) were recorded at 1000 Hz using the Delsys Trigno Avanti Sensors (Delsys, Boston, MA, USA). Data from the moment of initial ground contact to the moment of maximum knee flexion angle were processed for further analysis.

### Data Processing and Statistical Analysis

The OpenSim inverse kinematics tool (Stanford University, Stanford, CA, USA) was used to process the marker coordinates, which were low-pass filtered with a zero time-lag fourth order Butterworth filter at a cutoff frequency of 10 Hz to calculate the hip, knee, and ankle joint angles. The OpenSim inverse dynamics tool combined GRF and joint kinematic data to compute net internal joint moments. To ensure accuracy, force plate data were filtered similarly. The static optimization tool in OpenSim estimated individual muscle forces at each time step. Discrete Relative Phase (DRP) calculations assessed coordination between left and right limb joints for the hip (HDRP), knee (KDRP), and ankle (ADRP), respectively.

EMG data were processed with a Butterworth filter (cutoff frequencies 10 Hz and 450 Hz) to eliminate artifacts, and then fully rectified. After rectification, the signal was refiltered using a low-pass filter with a cutoff frequency of 5 Hz. Subsequently, the root mean square (RMS) amplitude and integrated EMG (IEMG) of the processed signal were calculated for each segment to determine the knee joint co-activation index (KCAI) and the ankle joint co-activation index (ACAI). To ensure the comparability of EMG data across different subjects, the Root Mean Square (RMS) amplitude values at each time point were normalized using the maximum value within the action cycle. This normalization ensured that the EMG data from different subjects were compared on the same scale. Additionally, left KAV and KS were computed. All calculation formulas for these variables are provided in the appendix.

Bland-Altman consistency evaluation plots were utilized to validate the musculoskeletal model's predictive capacity against actual surface EMG data. Statistical analysis was performed using SPSS 26.0 (SPSS, Chicago, IL, United States), with data expressed as mean ± standard deviation (SD). The Shapiro-Wilk test was first performed to assess data normality. Two-way repeated measures ANOVA was conducted to examine intervention effects. Post-hoc analyses were performed using Bonferroni correction to adjust for multiple comparisons. When the assumption of sphericity was violated, the Greenhouse-Geisser correction was applied to adjust the degrees of freedom. Effect sizes were reported as partial eta squared (η^2^) (small: 0.01 to 0.059, moderate: 0.06 to 0.137, and large > 0.138). For pairwise comparisons, the effect size was determined by Cohen's *d* with thresholds of 0.2 (small effect), 0.5 (medium effect), and 0.8 (large effect). The alpha level was set at *p* < 0.05 for significant difference, and *p* < 0.001 for highly significant difference.

Correlation analyses were conducted to identify variables correlated with PTF, including HDRP, KDRP, ADRP, KAV, KS, KCAI, ACAI, and IEMG contribution rates of individual muscles. Pearson correlation analysis and linear regression were employed to explore the relationships between the variables. Correlations were categorized as weak for coefficients between 0.01 and 0.16, moderate for coefficients between 0.16 and 0.49, strong for coefficients between 0.49 and 0.81, and very strong for coefficients between 0.81 and 1.00 (Schober et al., 2018). OPLS-DA was employed to distinguish between two group data and identify effective classification variables. Evaluation of the model predictive ability was done through the coefficient of determination for the Y variable (R2Y), cross-validated coefficient of determination (Q2Y), permutation testing for R2Y (pR2Y), permutation testing for Q2Y (pQ2), and Root Mean Squared Error of Estimation (RMSEE) values.

## Results

### Model Validation

**Figure 2:**
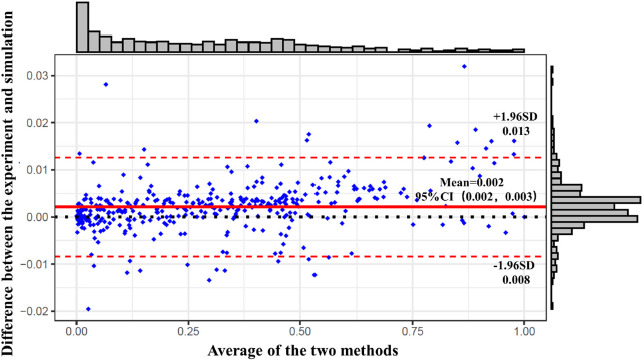
Bland-Altman consistency evaluation plot of the musculoskeletal model. Note: Red dashed lines represent a 95% confidence interval for the difference between measurement methods and red solid lines represent the average of the difference between the two measurement methods

The EMG acquisition data and OpenSim simulation data were normalized and subjected to a consistency assessment. In the Bland-Altman plot, the data form a horizontal band indicated no linear or a nonlinear relationship between the differences and the means. The histogram on the right side of Figure 2 shows that the differences follow a normal distribution. The results in Figure 2 demonstrate that the majority of the data points fall within the limits of agreement, indicating good consistency between the two methods and validating the reliability of our model.

### Discrete Relative Phase (DRP)

The interaction effect between groups and load accumulation on the ADRP was statistically significant (F(3, 24) = 1.677, *p* = 0.032). During the first three stages, the ASYM group exhibited significantly higher DRP values compared to the CON group, with differences of 7.34° (*d* = 0.351),11.24° (*d* = 0.728), and 2.49° (*d* = 0.187) of the absolute value, respectively (*p* < 0.05). Within the ASYM group, the DRP value during the 1^st^ quarter was significantly higher than during the 4^th^ quarter, with a difference of 1.53° (*p* < 0.05, *d* = 0.141).

### Kinetics and Muscle Activation Patterns

The interaction effect between groups and load accumulation on PTF was statistically significant (F(3, 24) = 4.128, *p* = 0.003). During the first three stages, the ASYM group exhibited significantly higher PTF values compared to the CON group, with differences of 0.98 (*d* = 0.791), 0.79 (*d* = 0.827), and 0.81 kg•BW^−1^ (*d* = 0.850), respectively (*p* < 0.001). In the CON group, PTF during the 4^th^ quarter was significantly higher than during the 3^rd^ quarter, with a difference of 0.22 kg•BW^−1^ (*p* < 0.05, *d* = 0.219).

The interaction effect between groups and load accumulation on KCAI was statistically significant (F(3, 12) = 8.033, *p* = 0.003). During the last three stages, the ASYM group exhibited significantly higher KCAI values compared to the CON group, with differences of 0.33 (*d* = 0.154), 0.28 (*d* = 0.257), and 0.25 (*d* = 0.635), respectively (*p* < 0.05). In the CON group, the KCAI during the 4^th^ quarter was significantly higher than during the 1^st^ quarter, with a difference of 0.26 (*p* < 0.05, *d* = 0.593).

### Relationships and Recognition among Variables

**Figure 3:**
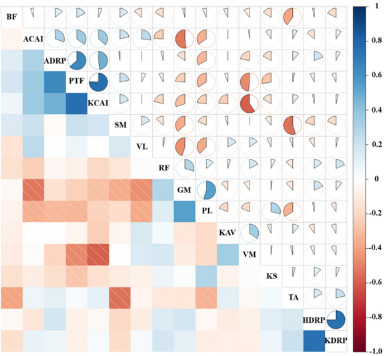
Detailed distributions of the correlation coefficient values between variables. PTF: patellar tendon force; HDRP: hip discrete relative phase; KDRP: knee discrete relative phase; ADRP: ankle discrete relative phase; KAV: left knee joint angular velocity; KS: knee joint stiffness; KCAI: knee joint co-activation index; ACAI: ankle joint co-activation index; BF, GM, PL, RF, SM, TA, VL, VM: IEMG contribution rates of each muscle

Figure 3 presents the detailed distribution of correlations between variables, depicting the results of data collection during landing. From Figure 3, it can be inferred that the highest correlations exist between PTF, the ADRP, and the KCAI, with correlation coefficients of 0.58 and 0.61, respectively, indicating a strong correlation.

**Figure 4:**
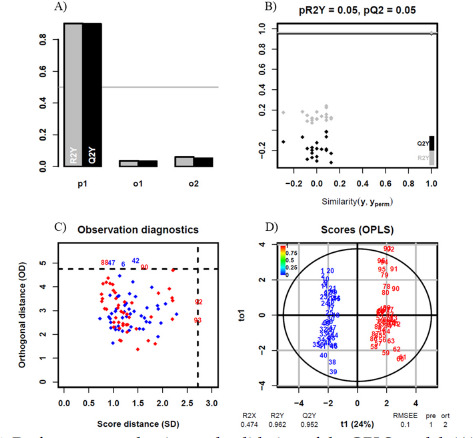
Performance evaluation and validation of the OPLS model; (A) model performance metrics; p1 indicates the model’s predictive component; o1 indicates the orthogonal component 1; o2 indicates the orthogonal component 2; (B) permutation test for model validation; (C) observation diagnostics; (D) scores plot from the OPLS model; blue indicates the ASYM group, and red indicates the CON group.

The OPLS-DA analysis results are shown in Figure 4. The R2Y(0.962) and Q2Y(0.952) values indicate good model performance in both fit and prediction. The models’ predictive component 1(p1), Orthogonal Component 1 (o1), and Orthogonal Component 2 (o2) further separate the variation related to the prediction from the unrelated systematic variation, helping the model better understand other secondary features in the data, as shown in Figure 4A. Figure 4B shows the distribution of R2Y and Q2Y values for the original model (shown as black dots) compared to permuted models (grey dots). pR2Y = 0.05 and pQ2 = 0.05 indicate the *p*-values for the permutation test, showing the significance of the R2Y and Q2Y values. Figure 4C displays orthogonal distance (OD) versus score distance (SD) for each observation. Red and blue dots represent the CON group and the ASYM group, respectively. Observations outside the dashed lines are potential outliers or influential points in the model. There is a small number of 7 outliers in total, which indicates the model's robustness. The OPLS scores plot shown in Figure 4D, indicates good separation between the sample groups, suggesting that the model can effectively distinguish between the conditions.

## Discussion

This study revealed significant differences in landing biomechanics between basketball players with and without ultrasound-detected patellar tendon structural abnormalities under game-specific simulated basketball loads. Notably, the PTF, ADRP, and KCAI showed marked differences. We also explored the relationships between various metrics and conducted classification modeling, which demonstrated strong interpretability and predictive capability.

Through this study, we found that the ASYM group exhibited significantly higher PTF and ADRP during landing in the first three stages and a higher KCAI in the last three stages. PTF was highly positively correlated with both the ADRP and the KCAI. This indicates that the lower limb joints in the ASYM group respond more intensely to the cumulative load, especially in the early stages of loading, which may increase the stress and risk of injury to the PT. Additionally, the classification of PT abnormalities based on the indicators proposed in this study demonstrated excellent predictive performance.

Previous studies have found that runners with abnormal tendon structures have an increased risk of experiencing pain within one year. Ultrasonography may be associated with a higher risk of subsequent tendon pain (Cushman et al., 2022; Kudron et al., 2020). In the event of asymptomatic ultrasound-detected tendon abnormalities, the relative risk of developing Achilles and PT pain is approximately three times higher (Cushman et al., 2021; Song et al., 2024). Therefore, we used ultrasound as a screening tool to select participants with asymptomatic PT abnormalities, allowing us to prospectively explore the factors leading to tendon structural abnormalities. Furthermore, we focused on guards in basketball and included cumulative game-specific simulated basketball loads to make the research more targeted and relevant to real conditions.

**Table 1:** DRP of joint couplings for the hip, knee, and ankle joints.

	ASYM				η^2^		F	*p*							
	1^st^	2^nd^	3^rd^	4^th^		1^st^			2^nd^		3^rd^	4^th^			
Knee DRP (°)	−9.64 ± 24.16	−13.62 ± 21.95	−8.22 ± 20.74	−16.38 ± 26.34		13.49 ± 48.97			−10.93 ± 65.11		13.94 ± 29.79	16.63 ± 32.43	0.005	0.037	0.866
Ankle DRP (°)	−48.81 ± 46.89^*^	−31.96 ± 73.01^*^	−44.74 ± 77.70^*^	−58.57 ± 81.75		−56.15 ±56.11 ^#*^			−43.20 ± 77.11^*^		−47.23 ± 87.38^*^	−63.68 ± 94.42^#^	0.259	1.677	0.032
Hip DRP (°)	−20.15 ± 30.47	−30.38 ± 25.21	−25.91 ± 26.27	23.89 ± 42.81		−13.49 ± 48.97			−11.78 ± 71.06		15.63 ± 29.33	18.50 ± 33.98	0.030	0.552	0.649
Knee Angular velocity (rad/s)	12.56 ± 1.71	12.16 ± 1.46	11.72 ± 0.61	12.85 ± 0.44		11.38 ± 1.21			11.78 ± 1.30		11.84 ± 0.90	11.56 ± 1.33	0.115	2.328	0.085

Note: ^*^ indicates a significant difference between the stages of the two groups; **^#^** indicates a significant difference between the stages within the same group

**Table 2:** Patellar tendon force, knee stiffness, and the joint co-activation index.

	CON				ASYM				η^2^	F	*p*
	1^st^	2^nd^	3^rd^	4^th^	1^st^	2^nd^	3^rd^	4^th^			
**Patellar tendon force (KG•BW^−1^)**	0.28 ± 0.20^*^	0.46 ± 0.29^*^	0.42 ± 0.23^*^^#^	0.64 ± 0.34 ^#^	1.26 ± 0.14^*^	1.25 ± 0.19^*^	1.23 ± 0.27^*^	1.24 ± 0.20	0.608	4.128	0.003
**Knee Stiffness (N∙m/°)**	0.59 ± 0.23	0.79 ± 0.49	0.75 ± 0.57	0.71 ± 0.47	1.27 ± 1.05	1.78 ± 1.22	1.48 ± 1.94	1.10 ± 1.97	0.281	2.082	0.143
**Knee joint co-activation index**	0.61 ± 0.16**^#^**	0.65 ± 0.07^*^	0.71 ± 0.34^*^	0.87 ± 0.02^*^^#^	0.78 ± 0.21	0.98 ± 0.19^*^	0.99 ± 0.30^*^	1.12 ± 0.23^*^	0.668	8.033	0.003
**Ankle joint co-activation index**	2.05 ± 0.63	1.89 ± 0.99	1.97 ± 0.79	0.93 ± 0.34	2.19 ± 0.87	2.39 ± 0.86	3.19 ± 1.32	1.91 ± 0.53	0.410	2.082	0.173

Note: ^*^ indicates a significant difference between the stages of the two groups; **^#^** indicates a significant difference between the stages within the same group

High PTF has been identified as a major contributing factor to patellar tendinopathy (Jie et al., 2024; Rudavsky and Cook, 2014). Our study further observed that basketball players with asymptomatic PT abnormalities exhibited higher PTF during the landing phase of stop-jumps. There was a notable interaction between the group classification and cumulative load affecting PTF. Specifically, the ASYM group demonstrated higher PTF levels in the initial three stages of load, particularly pronounced in the first stage. In contrast, within the CON group, PTF was higher in the fourth stage compared to the third, possibly due to cumulative fatigue effects. The heightened initial response of the ASYM group may stem from underlying structural abnormalities in their tendons, rendering them more vulnerable to stress. Our study also noted significant cumulative load effects on the knee KCAI, particularly evident in the CON group. This finding suggests that healthy athletes can effectively adjust muscle coordination strategies in response to progressively increasing loads to maintain knee joint stability. In contrast, individuals in the ASYM group, potentially affected by underlying knee joint issues, exhibited higher KCAI values during the early stages of load accumulation, possibly to cope with early physical demands and challenges. Previous studies on the results of the ADRP in volleyball-blocking landings have shown that females exhibit greater hip, knee, and ankle DRPs than males, indicating poorer lower limb symmetry during these movements. In our study, there was a significant interaction between group and cumulative load on the ADRP. The ASYM group showed higher ADRP values in the early stages, suggesting a stronger response to initial loads. However, this response diminished over time, possibly due to adaptation mechanisms or fatigue. Additionally, the ASYM group had greater absolute ADRP values than the CON group in the first three stages, indicating more asymmetrical loading on the passive support structures of the legs and poorer dynamic stability during landing (Hughes, 2014; Hughes and Watkins, 2008).

In a related study, Janssen et al. (2013) calculated PTF using knee joint torque and PT moment arm and predicted PTF through multivariate regression equations. They found that male participants with greater quadriceps strength, increased ankle dorsiflexion velocity, and increased trunk flexion velocity during landing were expected to generate higher PTF (Janssen et al., 2013). In our study, we collected surface EMG data and analyzed the activity status of relevant muscles, and the results showed significant differences in the effect of cumulative load on the KCAI between the ASYM and CON groups. Different response patterns in the KCAI were observed during cumulative loading. In the last three stages, the CON group had a lower KCAI, which may reflect a higher level of activation of knee joint agonist muscles compared to antagonist muscles. Viitasalo et al. (1998) found that trained long jumpers exhibited higher muscle activity in the gluteus medius, vastus lateralis, and gastrocnemius muscles during specific phases of jumping compared to untrained participants. Within the CON group, the increase in KCAI values from the 1^st^ to the 2^nd^ stage may indicate an increase in the activation level of knee joint antagonist muscles with cumulative load. This intra-group change suggests that individuals in the CON group respond to gradually increasing load during cumulative loading by enhancing muscle coactivation to maintain knee joint stability.

The correlation analysis among the test indicators revealed strong correlations: PTF with the ADRP (0.58) and the KCAI (0.61). This correlation between the ADRP and the KCAI with PTF may reflect the complex interactions between knee and ankle joints during jump landing, where coordination and stability influence muscle activity patterns and force transmission. During landing, the lower limbs experience a load pattern from distal to proximal, primarily controlled by the foot and the ankle, and then dispersed to the knee and hip joints (Zhang et al., 2018). The CON group exhibited better ankle joint symmetry, and early accumulation of load in the CON group showed better ankle joint symmetry, which may contribute to stabilizing the lower limb and reducing asymmetric loads borne by the knee joint, thereby reducing PTF. During single-leg landing, the ankle heavily relies on its surrounding muscular-tendon units to dissipate impact loads, particularly the ankle dorsiflexion, which provides 30%–50% of impact absorption (Lee et al., 2018). On the other hand, a higher KCAI indicates increased coactivation of muscles around the knee joint, potentially enhancing knee joint stability and control, but it may also increase the overall load on the knee joint. Therefore, the correlation between the ADRP, the KCAI, and PTF reveals the complex coordination mechanisms required by the lower limbs during landing to ensure the effectiveness and safety of movement. These findings emphasize the importance of focusing on ankle joint coordination and knee joint stability in sports training and rehabilitation. By optimizing the function of these joints, impact, and stress borne by the lower limbs during high-intensity activities can be better managed and alleviated, thereby preventing potential injuries.

Finally, we conducted classification modeling of the test indicators using OPLS-DA through machine learning methods. Previous studies have indicated that data-driven machine learning approaches are believed to perform modeling calculations faster than musculoskeletal models and have higher prediction accuracy. Additionally, Zou et al. (2022) developed a transfer learning model to estimate the knee contact medial force of knee valgus patients during rehabilitation gaits (Zou et al., 2022).

Janssen et al. (2013) utilized backward multiple regression analyses to determine which risk factors or landing technique variables were predictive factors for PT load. Xu et al. (2023) revealed a highly positive correlation between AIC, AROM, and PAF, and used the metaheuristic optimization algorithm SSA to optimize the model parameters. They proposed a secondary optimization by constructing a PAF prediction model, ultimately resulting in a highly accurate and easily implemented ACL force prediction model. Our classification model demonstrates high predictive capability (R2Y = 0.962, Q2Y = 0.952, pR2Y = 0.05, pQ2 = 0.05). Furthermore, we found that PTF, ADRP, KCAI, and IEMG contribution rates of VM play significant roles in discriminating between sample groups. In the future, these indicators are expected to be used for monitoring the PT health status of basketball players, facilitating monitoring during training or competitions, and aiding in the prevention of PT injuries.

This study focused exclusively on amateur basketball players without professional training experience. Therefore, the findings may not be directly generalizable to professional or elite-level basketball players. Additionally, some limitations could be optimized in future research. Firstly, our study only included male participants and focused on lower extremity indicators. Further research is needed to determine whether the patterns of PT load inferred from the results are universal. Additionally, discrepancies may still exist between simulated and actual basketball game loads.

## Conclusions

The study indicated that asymptomatic basketball players with PT abnormalities exhibited significantly higher PTF, ADRP, and KCAI after multiple game-specific simulated basketball load phases compared to healthy basketball players. This suggests that the increased mechanical load and stress response in the asymptomatic group may elevate the risk of patellar tendinopathy. To mitigate this risk, comprehensive strategies are necessary. Improving ankle joint symmetry during landing may help distribute lower limb forces more evenly, potentially reducing excessive stress on the PT. Additionally, optimizing strategies for the KCAI should focus on enhancing muscle coordination without compromising joint stability. Such measures can help decrease the mechanical load on the PT, thereby potentially lowering the incidence of patellar tendinopathy. Additionally, our classification model, which demonstrated strong interpretive and predictive capabilities, shows promise as a screening tool for monitoring PT health in athletes. In conclusion, by understanding the biomechanical differences and correlations between athletes with and without patellar tendinopathy, targeted interventions can be developed to prevent patellar tendinopathy, ultimately aiding in the maintenance of athlete performance and longevity in their sports careers.
